# The effect of prophylactic surgery in survival and HRQoL in appendiceal NEN

**DOI:** 10.1007/s12020-020-02356-8

**Published:** 2020-07-24

**Authors:** Krystallenia I. Alexandraki, Gregory Kaltsas, Simona Grozinsky-Glasberg, Kira Oleinikov, Beata Kos-Kudła, Angelika Kogut, Rajaventhan Srirajaskanthan, Michail Pizanias, Kalliopi-Anna Poulia, Clara Ferreira, Martin O. Weickert, Kosmas Daskalakis

**Affiliations:** 1 Endocrine Oncology Unit, 1st Department of Propaupedic Internal Medicine, Laiko Hospital, National and Kapodistrian University of Athens, Athens, Greece; 2 Neuroendocrine Tumour Unit, ENETS CoE, Endocrinology and Metabolism Department, Hadassah-Hebrew University Medical Center, Jerusalem, Israel; 3 Department of Endocrinology and Neuroendocrine Neoplasms, Department of Endocrinology and Pathophysiology, Medical University of Silesia, Katowice, Poland; 4 ENETS Centre of Excellence, Neuroendocrine Tumour Unit, King’s College Hospital, London, SE5 9RS UK; 5 Department of Gastroenterology, King’s College Hospital, London, SE5 9RS UK; 6 Department of Liver Transplantation, Hepatobiliary Pancreatic Surgery, King’s Healthcare Partners, King’s College Hospital, NHS FT, Institute of Liver Studies, Denmark Hill, London, UK; 7 Department of Nutrition, Laiko General Hospital, Athens, Greece; 8 Department of Nuclear Medicine, University Hospitals Coventry and Warwickshire NHS Trust, Coventry, UK; 9 The ARDEN NET Centre, European Neuroendocrine Tumour Society (ENETS) Centre of Excellence (CoE), University Hospitals Coventry and Warwickshire NHS Trust, Coventry, UK; 10 Clinical Sciences Research Laboratories, Warwick Medical School, University of Warwick, University Hospital, Coventry, UK; 11 Centre of Applied Biological & Exercise Sciences, Faculty of Health & Life Sciences, Coventry University, Coventry, UK; 12 Department of Surgery, Faculty of Medicine and Health, Örebro University, Örebro, Sweden

**Keywords:** Appendix, Carcinoid, Neuroendocrine tumor, Prophylactic surgery, Health-related quality of life

## Abstract

**Background/aims:**

Long-term outcomes are understudied in patients with well-differentiated appendiceal neuroendocrine neoplasms (WD-ANENs). We aimed to evaluate the validity of currently applied criteria for completion prophylactic right hemicolectomy (pRHC) and determine its association with patient outcomes, including health-related quality of life (HRQoL).

**Methods:**

Eligible patients from five European referral centers were divided between those who underwent appendectomy alone and those who underwent completion pRHC. HRQoL EORTC-QLC-C30 questionnaires and cross-sectional imaging data were prospectively collected. Age- and sex-matched healthy controls were recruited for HRQoL analysis’ validation.

**Results:**

We included 166 patients (119 women [71.2%]: mean age at baseline: 31 ± 16 years). Mean follow-up was 50.9 ± 54 months. Most patients (152 [92%]) had tumors ≤20 mm in size. Fifty-eight patients (34.9%) underwent pRHC that in final analysis was regarded as an overtreatment in 38/58 (65.5%). In multivariable analysis, tumor size >20 mm was the only independent predictor for lymph node (LN) involvement (*p* = 0.002). No mortality was reported, whereas 2-, 5- and 10-year recurrence-free survival in patients subjected to postoperative cross-sectional imaging (*n* = 136) was 98.5%, 97.8%, and 97.8%, respectively. Global HRQoL was not significantly impaired in patients with WD-ANEN compared with age- and sex-matched healthy individuals (median scores 0.83[0.08−1] vs 0.83[0.4−1], respectively; *p* = 0.929). Among patients with WD-ANEN impaired social functioning (*p* = 0.016), diarrhea (*p* = 0.003) and financial difficulties (0.024) were more frequently reported in the pRHC group.

**Conclusions:**

WD-ANEN is a low-malignant neoplasm with unconfirmed associated mortality, low recurrence rate, and overall preserved HRQoL. pRHC comes at a price of excessive surgery, functional HRQoL issues, and diarrhea. The value per se of a prophylactic surgical approach to patients with WD-ANENs <20 mm is challenged.

## Introduction

A worldwide increase in the incidence of neuroendocrine neoplasms (NENs) has recently been described, including appendiceal NENs (ANENs) [1]. This increment is most probably secondary to increased application of diagnostic procedures and widespread use of pathologic scrutiny of appendix specimens removed at appendectomy along with increased clinicians’ awareness. However, many of these patients might have remained untroubled by tumor-related consequences, had the tumor not been detected.

Well-differentiated ANENs (WD-ANENs) are mainly of Grade 1 and commonly exhibit a benign clinical course with only few cases being reported with distant metastases [2, 3]. Therefore, ANEN standard treatment is surgery, which can be limited to the initial appendectomy or completed with a prophylactic right hemicolectomy (pRHC), with the latter potentially been associated with adverse consequences in terms of long-term health-related quality of life (HRQoL) impairment [4]. Contemporary guidelines suggest a rather aggressive surgical approach with completion pRHC as a treatment option for patients diagnosed with tumors ≥20 mm or tumors 10–20 mm in the presence of certain histopathological parameters [5, 6]. However, recent cohort studies and meta-analyses have questioned the validity of currently applied criteria for completion pRHC in WD-ANENs [2, 7–10]. Nevertheless, the association of indolent locoregional lymph node (LN) metastases with long-term patient outcomes has not been fully determined [10, 11]. Hence, a more conservative strategy with an active surveillance approach following appendectomy to WD-ANEN patients with histopathological risk parameters has only lately been suggested pending confirmation from prolonged follow-up data [2, 12, 13].

Importantly, patient outcomes have been assessed in ANEN clinical studies, using mainly parameters, such as LN positivity at completion pRHC. However, apart from the yet undetermined survival benefit of a prophylactical surgical approach to these patients, a more comprehensive assessment of HRQoL aspects and prospective imaging confirmation of disease status is largely missing. In the era of a “less is more” approach and the possibility of performing likely unnecessary surgeries in patients with WD-ANENs, this study aimed to evaluate the rate of potentially unnecessary pRHCs in WD-ANEN patients and the validity of contemporary criteria. In addition, we aimed to evaluate whether certain histopathological parameters applied for completion surgery could predict locoregional LN status at pRHC; to determine the prognostic impact of LN metastases with regards to recurrence and survival outcomes; and to assess the range of HRQoL outcomes, and whether these vary depending on the type of surgery the patient received.

## Methods

### Study design

Data were extracted from the NEN databases of five collaborating European Neuroendocrine Tumor Society (ENETS) Centers of Excellence: the EKPA-Laiko Center, Athens, Greece; the ARDEN NET Center, University Hospitals Coventry and Warwickshire, NHS Trust, Coventry, UK (audit number 515/2019); the Neuroendocrine Tumor Unit, KHP ENETS Center of Excellence, King’s College Hospital, London, UK; the Neuroendocrine Tumor Unit, Endocrinology and Metabolism Department, Hadassah–Hebrew University Medical Center, Jerusalem, Israel; and the Department of Endocrinology and Neuroendocrine Neoplasms, Department of Endocrinology and Pathophysiology, Medical University of Silesia, Katowice, Poland. We included only patients with a definite histopathological WD-ANEN diagnosis, with disease stage I–III. Patients with goblet cell appendiceal tumors and tumors of mixed histopathology were excluded. In addition, patients operated upfront with more extensive bowel resections (small bowel resection, caecectomy, right hemicolectomy, or total colectomy) than appendectomy alone at the time of incidental WD-ANEN diagnosis for any indication (malignancy or benign conditions) were not included in this study.

The diagnoses were made between the 1st of August 1992 and the 31st of July 2019, and patients were followed until death or September 15th, 2019. Cross-sectional imaging with abdominal magnetic resonance imaging (MRI) was prospectively obtained in patients with follow-up >10 years. As part of the study, participants were also asked to fill in a HRQOL EORTC-QLQ-C30 questionnaire [14]. HRQOL questionnaires were also obtained from 20 healthy control cases of age- and sex-matched individuals who did not have any abdominal surgery in their personal history, for comparison.

The study was conducted according to the 1975 Declaration of Helsinki and approved by the pertinent Human Research Ethics Committee of each institution. In the collaborating centers from UK, the study was officially registered as an audit. Written informed consent for the study was obtained from study participants. To ensure the quality of data reporting, we followed the STROBE statement [15].

At diagnosis, patients underwent cross-sectional imaging with either computed tomography (CT) scan or magnetic resonance imaging (MRI) of the abdomen. Each center followed the ENETS guidelines for completion pRHC following incidental diagnosis at appendectomy [5]. Subsequently, surveillance protocols with sequential cross-sectional imaging of the abdomen with computed tomography (CT) or MRI were performed to assess disease recurrence (locoregional and/or at distant sites-mainly the liver) in lymph node positive WD-ANEN patients subjected to pRHC and also patients at high risk (i.e. tumor size ≥20 mm, ANEN located in appendix base, grade 2, mesoappendiceal-, vascular-, lymph vessel-, and/or perineural invasion) not subjected to pRHC for reasons including other comorbidities and no consent, as per ENETS guidelines [5]. Due to cumulative exposure to irradiation with repetitive CT scanning, MRI was performed in most patients, particularly in younger and of fertile age [16]. In addition, although unproven, taken into consideration the life-long awareness of the potential of recurrence for WD-ANEN > 20 mm or >10 mm with risk factors, different surveillance approaches with MRI at varying intervals were applied at each center also for patients who underwent a pRHC [5, 17]. Tumor grade was determined from primary appendix specimens according to the Ki-67 proliferation index by a dedicated histopathologist at each center according to ENETS guidelines [5]. We used the 2017 WHO classification systems for grading gastro-enteropancreatic NENs [18]. For staging, we used the 8th edition of the American Joint Committee on Cancer (AJCC) [19].

We used the EORTC QLQ-C30 questionnaire developed by the European Organization for Research and Treatment of Cancer (EORTC) that is composed of multi-item functional subscales: physical, role, emotional, social and cognitive functioning; three multi-item symptom scales measuring fatigue, pain, and emesis; global health/quality of life subscale; and six single items assessing financial impact and symptoms such as dyspnea, sleep disturbance, appetite, diarrhea, and constipation [14]. Patients’ responses were evaluated and summarized according to standard HRQoL nomenclature [20].

After data review, patients were divided between those who underwent appendectomy alone (Appendectomy group) and those who underwent completion pRHC (pRHC group). To avoid immortal time bias, baseline for the pRHC group was defined as the first date on which patients underwent pRHC, whereas baseline for the Appendectomy group was the date of appendectomy.

### Statistical analysis

Variables are presented as medians with ranges or means with standard deviations (SDs), as appropriate. Differences between groups were assessed using the Chi-square test and the non-parametric Mann–Whitney test, as appropriate, due to the non-normality of data distributions, but also the ordinal nature of the patient-based HRQoL to limit assumptions and maintain consistency. Multivariable logistic regression analysis was performed to assess histopathological parameters associated with LN positivity at pRHC. Quantitative HRQoL data analysis was conducted to explore whether the type of surgical procedure (appendectomy vs pRHC) was associated with the reporting of HRQOL issues. All tests were 2 sided unless stated otherwise. *P* < 0.05 was considered to be significant for all tests. All statistical analyses were done with the SPSS v23.0 software package (IBM SPSS Statistics, Armonk, NY, USA).

## Results

**Fig. 1:**
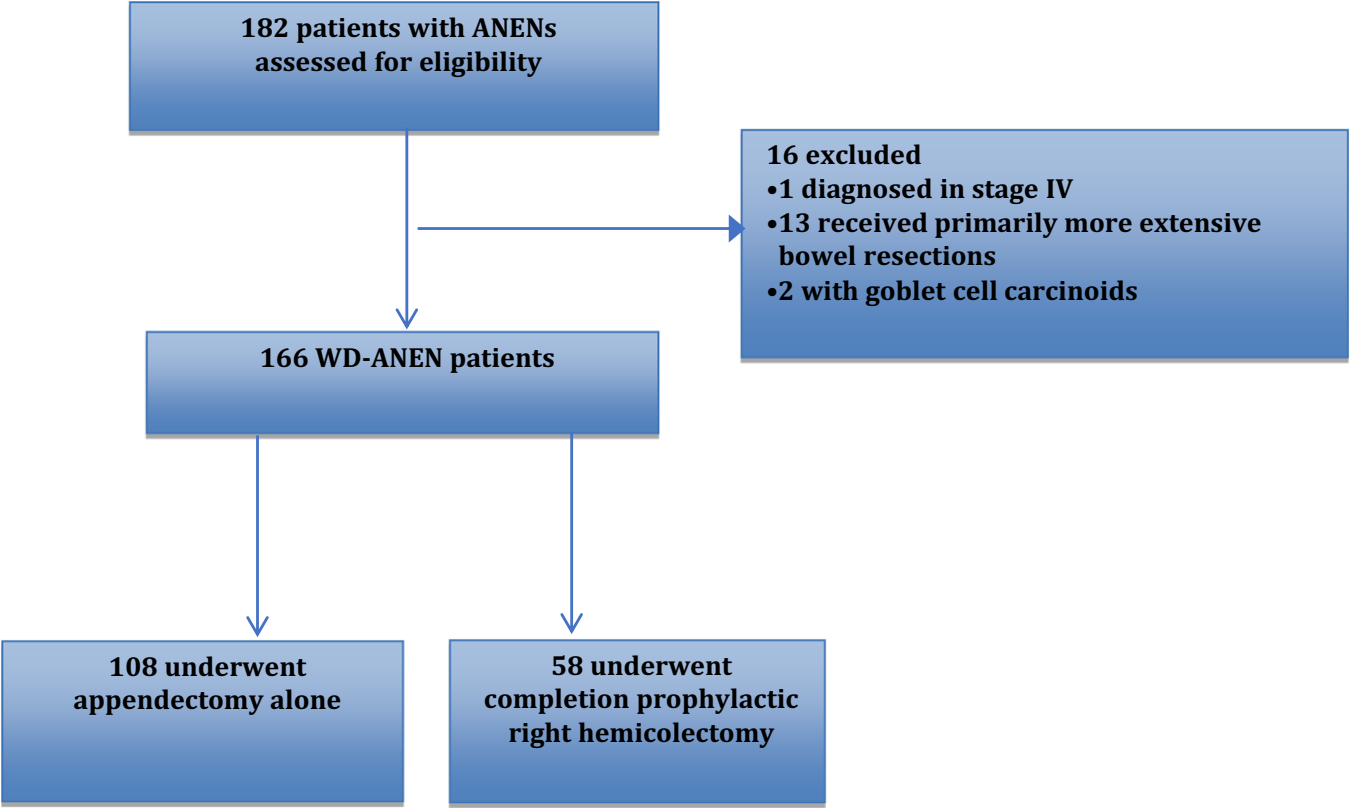
Study flow chart

Of a total of 182 patients with ANENs, 166 patients fulfilled the inclusion criteria (119 women [71.2%]) and 47 men [28.8%]). Mean (±SD) age at baseline was 31 (±16) years and most patients were otherwise healthy (Charlson comorbidity index ≤1 in 154/166 [92.8%] patients; Table 1) [21]. Mean (±SD) overall follow-up was 50.9 (±54) months. One hundred eight patients had appendectomy alone (Appendectomy group; *n* = 108), whereas 58 (34.9%) underwent completion pRHC following primary appendectomy (pRHC group; *n* = 58; mean(±SD) time to completion: 116,4 ± 121.9 days) (Fig. 1). Most patients (149[89.8%]) had tumors <20 mm in size and 71 (42.8%) had tumors in the zone of 10–20 mm. In the pRHC group, 43 patients had tumors ≤20 mm, whereas 39 patients in the appendectomy group had tumors in the gray zone of 10–20 mm (Table 1).

**Table 1:** Clinicopathological characteristics of patients diagnosed with well-differentiated appendiceal neuroendocrine neoplasms included in the study cohort at initial diagnosis (*n* = 166)

Patient and tumor characteristics	Number of patients (%)
Mean age (±SD)*	31 (±16)
Gender	
Female	119 (71.2)
Male	47 (28.8)
Type/extent of surgery	
Appendectomy alone	108 (65.1)
Completion pRHC	58 (34.9)
Charlson comorbidity Index	
0	138 (83.1)
1	16 (9.6)
2	7 (4.2)
3	2 (1.2)
≥4	2 (1.2)
Tumor size	
<1 cm	74 (44.6)
1–2 cm	71 (42.8)
>2 cm	16 (9.6)
unknown	5 (3)
Grade	
G1	144 (86.7)
G2	18 (10.8)
Unknown	4 (2.4)
Location	
Base	18 (10.8)
Body	28 (16.9)
Apex	91 (54.8)
Unknown	19 (11.5)
Mesoappendiceal invasion	
No	86 (51.8)
Yes	76 (45.8)
Unknown	4 (2.4)
Vascular invasion	
No	143 (86.1)
Yes	19 (11.4)
Unknown	4 (2.4)
Lymph vessel invasion	
No	147 (88.6)
Yes	15 (9)
Unknown	4 (2.4)
Perineural invasion	
No	140 (84.3)
Yes	22 (15.3)
Unknown	4 (2.4)

*At baseline

Completion pRHC was likely unecessary in 38/58 patients (65.5%; no residual disease or locoregional LN metastases); and in 34/43 patients (79.1%) in the subset of patients with tumors ≤20 mm subjected to prophylactic surgery. On the other hand, 13/39 patients (33.3%) with tumor size 10–20 mm had one or more risk factors for positive LN status (such as grade 2, vascular-, lymph vessel- and/or perineural invasion), but underwent appendectomy alone. Among the investigated clinicopathological parameters used for completion pRHC, tumor size ≥20 mm was confirmed as an independent prognostic factor for LN positivity on multivariable logistic regression analysis (HR = 0.058; 95%CI:0.009–0.351; *p* = 0.002; Table 2). Neither overall- nor disease-specific mortality was encountered in this series. Three patients developed a recurrence (*n* = 2 in the pRHC group vs *n* = 1 in the Appendectomy group; *p* = 0.263). Among the two patients in the pRHC group who developed recurrence, one had positive LN at diagnosis, whereas the patient in the Appendectomy group with a recurrence had no risk factors. Two of the patients developed distant metastases found at cross-sectional imaging; one to the liver (pRHC group) and one to the bones (Appendectomy group), whereas one to locoregional LN (metachronous LN metastases; pRHC group). Importantly, none among the 13 patients in the appendectomy group with tumors 10–20 mm in size and one or more risk factors had residual disease or developed a recurrence at subsequent cross-sectional imaging.

**Table 2:** Binary logistic regression model with risk assessment for positive lymph node status at completion prophylactic right hemicolectomy in patients with well-differentiated appendiceal neuroendocrine neoplasms (*n* = 58) included in this study

Prognostic factor for positive LN status at pRHC (*n* = 58)			
	HR	95%CI	*P* value
Tumor size			**0.002**
≥20 mm	1		
<20 mm	0.058	0.009–0.351	
Grade			0.328
G1	1		
G2	3.68	0.271–49.89	
Location			0.473
Base	1		
Non-base	0.518	0.086–3.119	
Mesoappendiceal invasion			0.525
No	1		
Yes	1.762	0.307–10.117	
Vascular invasion			0.853
No	0.786	0.061–10.140	
Yes	1		
Lymph vessel invasion			0.091
No	0.090	0.06–1.469	
Yes	1		
Perineural invasion			0.137
No	0.208	0.26–1.652	
Yes	1		

Bold value indicates statistically significant

**Fig. 2:**
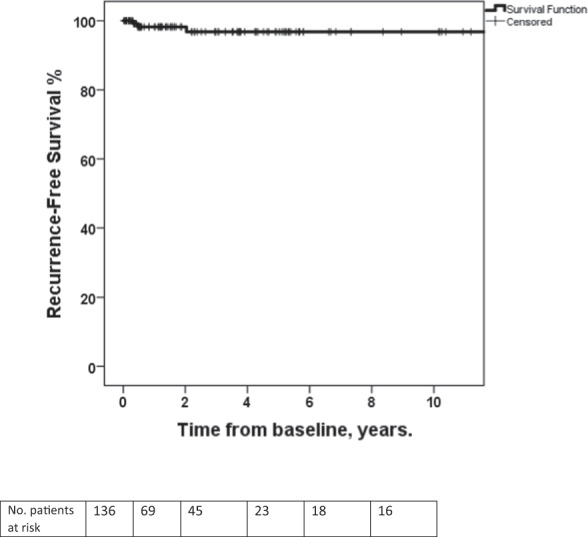
Recurrence-free survival in patients with well-differentiated appendiceal neuroendocrine neoplasms subjected to postoperative cross-sectional imaging (*n* = 136), commonly magnetic resonance tomography. Survival estimates assessed from baseline

Overall, 136 WD-ANEN patients (81.9%) were subjected to postoperative cross-sectional imaging with the remaining 30 patients being discharged after surgery. In particular, 69 patients (50.7%) had an MRI scan or less commonly a CT scan performed more than 2 years from baseline, whereas in 38 (22.9%) and 16 patients (9.6%) cross-sectional imaging was obtained more than 5- and 10-years from baseline, respectively. Two-, five- and ten-year recurrence-free survival (RFS) in patients subjected to cross-sectional imaging (*n* = 136) was 98.5%, 97.8% and 97.8%, respectively (Fig. 2).

With regards to other treatments, three patients in this series received somatostatin analogues after initial surgery (two patients due to recurrence). Finally, one additional patient with WD-ANEN from the initial cohort presented with liver metastasis at diagnosis and therefore was excluded from the analysis (3/180 patients with stage IV disease at any point [1.7%]; Fig. 1). Two patients in the pRHC group (2/58; 3.4%) were re-operated subsequently for bowel obstruction due to postoperative adhesions, whereas re-operations due to incisional hernias were not reported during the study follow-up.

The following HRQoL issues emerged from content analysis of 79 patient responses (any change in global HRQoL/symptom scales vs none): global health status (64[81%]), physical-(41[51.2%]), role-(23[29.1%]), emotional-(48 [60%]), cognitive-(32 [40.5%]) and social functioning (25[31.6%]). Global HRQoL was not significantly depreciated in patients with WD-ANEN (*n* = 79) compared with age- and sex-matched healthy individuals without any abdominal surgery at their medical history (median scores[range]: 0.83[0.08–1] vs 0.83[0.4–1], respectively; *p* = 0.929). However, appetite (*p* = 0.040) and diarrhea (0.017) concerns were more often reported among WD-ANEN patients than healthy controls (Table 3). Among WD-ANEN patients that participated in the HRQoL assessment, global HRQoL was not significantly impaired in patients undergoing pRHC compared with appendectomy alone (median scores 0.79[0.25–1] vs 0.83[0.08–1], respectively; *p* = 0.738). In addition, the multivariable logistic regression model (adjusted for age and sex) applied for HRQoL analysis, did not provided any evidence of a difference in reporting of a change in or concern about global HRQoL (any vs none) between surgery types in the two groups of the study (pRHC group vs App group; HR = 0.768; 95%CI: 0.234–2.518; *p* = 0.663). However, analyses in functional and symptom scales revealed that impaired social functioning (*p* = 0.016), diarrhea (*p* = 0.003), and financial difficulties (0.024) were more frequently reported in the pRHC group (Table 4). Furthermore, physical- (*p* = 0.066) and role functioning (*p* = 0.055), as well as constipation issues (*p* = 0.072) emerged in the pRHC group with marginal significance (Table 4). Diarrhea was mainly attributed to pRHC as further comparison between the Appendectomy group and the group of healthy controls did not yield any statistical significance on this particular outcome (median scores[range]: 0[0–0.33] vs 0[0–0.67]; *p* = 0.141). Comparable number of patients in the pRHC and appendectomy group fulfilled the HRQoL questionnaires (49/108 [45.4%] vs 30/58[51.7%], respectively).

**Table 3:** Comparison of Health-Related Quality of Life concerns between patients with well-differentiated appendiceal neuroendocrine tumors (*n* = 79) and age- and sex-matched individuals without any abdominal surgery at own medical history (*n* = 20)

HRQoL issue	Median scores (95%CI) in WD-ANEN patients (*n* = 79)	Median scores (95%CI) in matched controls (*n* = 20)	*P* value
Global HRQoL	0.83 (0.08–1)	0.83 (0.4–1)	0.929
Functional issues			
Physical functioning	1 (0.86–0.94)	0.97 (0.91–0.97)	0.910
Role functioning	1 (0.87–0.95)	1 (0.82–0.96)	0.318
Emotional functioning	0.83 (0.72–0.84)	0.88 (0.71–0.9)	0.989
Cognitive functioning	1 (0.82–0.91)	1 (0.76–0.99)	0.436
Social functioning	1 (0.82–0.92)	1 (0.83–0.99)	0.596
Symptom scales			
Fatigue	0.22 (0.21–0.34)	0.33 (0.25–0.46)	0.117
Nausea	0 (0.03–0.11)	0 (−0.01–0.06)	0.202
Pain	0 (0.11–0.20)	0 (0.03–0.16)	0.286
Dyspnoea	0 (0.03–0.16)	0 (0.003–0.13)	0.740
Insomnia	0 (0.17–0.32)	0 (0.01–0.29)	0.641
Appetite	0 (0.10–0.219	0 (−0.01–0.08)	**0.040**
Constipation	0 (0.07–0.18)	0 (0.01–0.27)	0.298
Diarrhea	0 (0.13–0.25)	0 (−0.01–0.11)	**0.017**
Financial difficulties	0 (0.06–0.18)	0 (−0.02–0.15)	0.389

Bold values indicates statistically significant

**Table 4:** Comparison of Health-Related Quality of Life concerns between patients with well-differentiated appendiceal neuroendocrine neoplasms (WD-ANEN) undergoing appendectomy alone (*n* = 49) and WD-ANEN patients undergoing completion prophylactic right hemicolectomy (*n* = 30)

HRQoL issue	Median scores (95%CI) in Appendectomy group (*n* = 49)	Median scores (95%CI) in pRHC group (*n* = 30)	*P* value
Global HRQoL	0.83 (0.68–0.81)	0.79 (0.64–0.81)	0.738
Functional issues			
Physical functioning	1 (0.87–0.96)	0.93 (0.80–0.94)	0.066
Role functioning	1 (0.91–0.98)	1 (0.76–0.95)	0.055
Emotional functioning	0.83 (0.71–0.85)	0.92 (0.68–0.88)	0.907
Cognitive functioning	1 (0.82–0.94)	1 (o.76–0.93)	0.443
Social functioning	1 (0.85–0.97)	1 (0.70–0.90)	**0.016**
Symptom scales			
Fatigue	0.22 (0.17–0.28)	0.33 (0.22–0.48)	0.182
Nausea	0 (0.02–0.08)	0 (0.02–0.19)	0.407
Pain	0 (0.07–0.17)	0.17 (0.12–0.29)	0.137
Dyspnoea	0 (0.01–0.13)	0 (0.02–0.17)	0.290
Insomnia	0 (0.14–0.3)	0 (0.15–0.44)	0.406
Appetite	0 (0.09–0.22)	0 (0.03–0.28)	0.415
Constipation	0 (0.03–0.15)	0 (0.08–0.3)	0.072
Diarrhea	0 (0.06–0.17)	0.33 (0.2–0.42)	**0.003**
Financial difficulties	0 (0.08–0.32)	0 (0.02–0.17)	**0.024**

Bold values indicates statistically significant

## Discussion

In the present study, we assessed in a multicenter setting, outcomes of patients with WD-ANENs after completion pRHC compared with appendectomy alone. A prophylactic surgical approach based on contemporary criteria in this series suggested that pRHC was probably an overtreatment in approximately two-thirds of the patients undergoing pRHC. Appendectomy alone would have been a curative measure in 84% of all patients and in 92.5% of patients with tumors <20 mm in size. Among patients selected for completion pRHC with tumors <20 mm in size and one or more risk factors favouring a pRHC, approximately one fifth will be found to have LN metastases at reoperation. In addition, no mortality and very low recurrence rate were evident in our study, questioning the prognostic impact of LN involvement at pRHC. Most WD-ANEN patients in this series (81.9%) were subjected to postoperative cross-sectional imaging, commonly MRI, and exhibited favorable RFS figures at 2-, 5- and 10-year RFS as high as 98.5%, 97.8%, and 97.8%, respectively. Although we found no difference in global HRQoL between WD-ANEN patients and age-/sex-matched healthy controls, impaired social functioning, diarrhea, and financial difficulties were more frequently reported in patients undergoing completion pRHC. Furthermore, physical and role functioning, as well as constipation issues emerged in the pRHC group with marginal significance.

Traditionally, a tumor size >20 mm constitutes a general indication for completion pRHC based on the seminal paper in the field by Moertel et al. [22]. This parameter was subsequently validated in numerous studies and recent meta-analyses and also confirmed in the multivariable logistic regression analysis of the present study [2, 5, 8, 9, 23–26]. ENETS guidelines have also considered tumor size >20 mm to guide the extent of surgery in patients with WD-ANENs. For tumor size 10–20 mm, various histopathological parameters have been applied to identify right candidates for completion pRHC although not fully validated. In particular, the location of the primary tumor in the base of the appendix, grade 2, mesoappendix, vascular, lymph vessel, and perineural invasion has been implied as a risk factors for synchronous LN metastases necessitating completion pRHC [7, 8, 13, 27, 28]. However, these factors were not confirmed in our study with respect to LN metastases prediction. The reasons that our data could not confirm the role of certain histopathological parameters, for example that of lymphovascular invasion to predict nodal involvement may be multifactorial, including lack of centralized pathology review, but also limitations inherent to the nature of databases, such as the Surveillance, Epidemiology, and End Results Program (SEER) and the National Cancer Database (NCDB) with unrecorded variables, underreported and incomplete data, NEN heterogeneity and finally the inclusion of goblet cell appendiceal tumors and tumors of mixed histopathology in other studies.

Several recent studies have implied a low-malignant tumor biology and challenged the clinical relevance of indolent positive LN at pRHC as it appears to exert no major effect on overall survival [2, 12, 13, 27, 29, 30]. However, to date there is no definite data regarding the long-term outcomes of this patient group. Three patients only in our series demonstrated disease recurrence, two of whom underwent a pRHC according to ENETS guidelines. Overall, the prevalence of stage IV disease at any point in the initial cohort was as low as 1.7% whereas all patients were alive at last follow-up, in line with other recent studies [2, 31]. The 50-month mean follow-up period of patients in this cohort and the relatively small subset of patients followed longer than 5- and 10-years respectively may limit our conclusions on long-term patient outcomes. However, this follow-up is one of the longest reported in WD-ANEN patients after correction for potential immortal time bias. Cross-sectional imaging confirmation of disease status in most patients (81.9%) was one of the main strengths of this study as well as the availability of imaging data in the small subset of patients with risk factors who did not have pRHC and also that of prospective MRI data in patients with follow-up >10 years.

With regards to HRQoL analysis, the reliability and validity of the employed HRQoL instruments in WD-ANEN patients assessing the treatment option of pRHC may be more straight-forward, than in other aggressive cancer forms where somatic manifestations of psychological distress might be attributed to the tumor itself rather than the treatment of it. As only three patients developed recurrence and no systemic treatments were given to almost all WD-ANEN patients, HRQoL differences between the Appendectomy- and the pRHC group could be mainly attributed to the type/extent of the surgical procedure undertaken.

This study has some limitations, including the retrospective nature of the risk assessment analysis for LN metastases and the lack of centralized pathology review that mainly concerns interstudy concordance for tumor size and that of other histopathological parameters. However, histopathological review was conducted by dedicated specialists in the involved ENETS centers and prospectively collected MRI data were used to accurately define disease status in the long-term. In addition, as our analysis was based on data from patients referred to specialized centers, referral bias might have skewed the results. Although not all patients of this cohort participated in the HRQoL analysis, a significant number from each group fulfilled the HRQoL questionnaires. Nevertheless, long-term evaluation mainly with MRI at 5 and 10 years of follow-up was available for a minority of patients, thus inferences on long-term recurrence should be interpreted with caution. The results from multivariable analysis on a relatively rare tumor entity suggests only size >20 mmm is a significant factor predicting abnormal post-operative pathology; therefore, other features such as tumor location in the base of the appendix, grade 2, mesoappendix, vascular, lymph vessel, and perineural invasion in 1–2 cm lesions should not be indications for completion pRHC. In addition, functional HRQoL concerns and diarrhea may affect a substantial proportion of patients undergoing prophylactic surgery. To our knowledge, this study is the first to analyze HRQoL issues among patients with WD-ANEN and among few that assess long-term outcomes in this patient group.

In conclusion, our study demonstrates that patients with WD-ANENs whose surgical management is based on contemporary criteria for a prophylactic surgical approach are subjected to excessive surgery and exhibit functional HRQoL concerns and development of diarrhea. In light of our results, the value of prophylactic surgery should probably be reconsidered and the appropriateness of a more conservative approach at least in tumors <20 mm in size should be employed. As reliable biomarkers allowing targeted preventive surgery are not currently available in WD-ANENs, appendectomy alone may be as safe in terms of survival outcomes in almost all patients and could lead to a reduction of overtreatment and related adverse effects. In the era of personalized medicine, ANEN patients and involved physicians should consider the implications of WD-ANEN diagnosis as a low-malignant entity and the implementation of appropriate surveillance strategies to balance any negative surgery-related outcomes.
